# Efficacy of Ascaroside #18 Treatments in Control of *Salmonella enterica* on Alfalfa and Fenugreek Seeds and Sprouts

**DOI:** 10.1016/j.jfp.2023.100064

**Published:** 2023-02-11

**Authors:** Xueyan Hu, Seulgi Lee, Murli Manohar, Jinru Chen

**Affiliations:** 1 Department of Food Science and Technology, The University of Georgia, Griffin, GA 30223-1797, USA; 2 Ascribe Bioscience, Ithaca, NY 14850, USA

**Keywords:** Ascr#18, Salmonella enterica, Sprout seeds, Vegetable sprouts, Plant immunity modulator

## Abstract

A novel, natural, and effective antimicrobial intervention is in demand for improving the microbial safety of vegetable seeds/sprouts. This study assessed the efficacy of ascaroside treatment in the control of *Salmonella enterica* on alfalfa and fenugreek sprouts. Sanitized commercial seeds were treated with 1 mM or 1 μM ascaroside (ascr)#18, a plant immunity modulator (PIM) and dried for an hour before being inoculated with lyophilized *S*. Cubana or *S*. Stanley cells in sandy soil (10^4^ CFU/g). Treated and untreated seeds were spouted on 1% water agar at 25°C in the dark. Seed or sprout samples were collected on days 0, 1, 3, 5, and 7, and the population of *Salmonella* was determined. Data were fit into the general linear arrangement, and means were separated using Fisher’s least significant difference test. Seed type, strain type, treatment type, and sprouting time were significant factors (*P* ≤ 0.05) influencing *Salmonella* growth on sprouts. The populations of *Salmonella* were significantly higher on fenugreek than on alfalfa sprouts. *S*. Stanley had a significantly higher population than *S*. Cubana. The population of *Salmonella* increased from day 0 to day 3 and reached the peak population on Day 5. Treatments with both concentrations of ascaroside significantly decreased the populations of *Salmonella* compared to the controls. The mean *Salmonella* population reduction was *ca*. 4 or 1 log CFU/g by treatment with 1 mM and 1 μM of the PIM, respectively. Treatment with the PIM could be potentially used to improve the microbial safety of vegetable seeds and sprouts.

Sprouts are young shoots that are germinated from seeds. Commonly consumed sprouts include those produced from alfalfa or fenugreek seeds. Alfalfa (*Medicago sativa*) is a flowering plant in the pea family (*Fabaceae* or *Leguminosae*) (Hong et al., 2011), and its sprouts are often added to sandwiches and salads. The consumption of alfalfa sprouts has increased in recent years because modern consumers prefer foods that are more nutritious and subjected to less processing (Miyahira et al., 2021; Frías et al., 2007). Fenugreek (*Trigonella foenum-graecum*) is also from the *Fabaceae* family. It has been used not only as a food source but also as a therapeutic agent since fenugreek contains active substances such as alkaloids, flavonoids, steroids, and so on. Thus, fenugreek sprouts are considered healthy and diseasepreventing (Snehlata & Payal, 2012). During the germination of seeds, many macronutrients and antinutrients are metabolized, and secondary metabolic compounds are synthesized, making germinated sprouts have potential health benefits (Peñas & Martínez-Villaluenga, 2020).

The commercial sprouting process involves seed rinse, seed treatment, pregermination seed soak, germination and growth, harvest, wash/drain sprouts, bulk cool/spin, dry pack and/or package, cooling and storage, as well as distribution (US HHS and FDA, 2017). Some of these processes provide favorable environmental conditions and abundant nutrition to support the growth of foodborne pathogens (Ding et al., 2013); thus, leafy sprouts are easily contaminated by them during the production process. Furthermore, sprouts are usually consumed raw or lightly cooked and there is no killing step before consumption (Proctor et al., 2001). As a result, sprout-related outbreaks of gastrointestinal infection occur quite frequently. According to the Centers for Disease Control and Prevention, there were at least 64 outbreaks related to sprouts from 1988 to 2020 worldwide, including 46 outbreaks in the United States (Miyahira et al., 2021). Alfalfa sprouts were involved in more than 55% of these outbreaks. Although in 1999, the U.S. Food and Drug Administration recommended the use of 20,000 ppm calcium hypochlorite to decontaminate sprout seeds before germination, sprout-associated outbreaks continued to occur (Winthrop et al., 2003). Thus, more effective, alternative treatments are in demand.

It was found recently that low concentrations of ascr#18 (referred as the plant immunity modulator thereafter [PIM]), a major component of the ascarosides secreted by plant-parasite nematodes, could act as a pheromone to boost plant resistance to viral, bacterial, oomycete, fungal, and nematode infections by triggering their immune responses (Manosalva et al., 2015). Ascarosides are derivatives of dideoxy sugar combined with fatty acid-derived lipophilic side chains or other primary metabolism-derived moieties (Klessig et al., 2019). They cannot be defined as pesticides or pathogen-cides because they do not kill pests and pathogens (Bouchie, 2019). The PIM is a major component of a conserved family of nematode pheromones. It is perceived by plant cells, when encountered, as pathogen invasion which triggers by something like the so-called “microbe or pathogen-associated molecular patterns” (MAMPs or PAMPs), subsequently leading to the activation of plant immune responses. MAMPs or PAMPs are specific molecules or parts of certain molecules that have conserved structures or chemical patterns unique to a specific pathogenic microorganism. Lipopolysaccharides, peptidoglycan, and flagellin of bacterial pathogens are MAMPs to human cells during infection (Erbs & Newman, 2012). These molecules or patterns can be recognized by specific pattern recognition receptors on the surface of host cells. Ascarosides could trigger a similar immune response by the “nematode-associated molecular patterns” (NAMPs) (Choi & Klessig, 2016). The activated characteristic plant responses include the following: 1). The onset of conserved signal transduction through upregulation of the salicylic acid and jasmonic acid pathways (Pieterse et al., 2012); 2). Activation of certain enzymes, such as mitogen-activated protein kinase and calcium-dependent protein kinase, which are important actors of plant signaling that can elicit a wide range of physiological responses in the cells (Ichimura & Mapk Group, 2002; Yip Delormel & Boudsocq, 2019), and 3). Production of reactive oxygen species during the process, which makes plants maintain normal growth and improve tolerance to stress (Huang et al., 2019).

The objective of this study was to investigate the effectiveness of the PIM treatment against *Salmonella enterica* on alfalfa and fenugreek sprouts during the sprouting process.

## Materials and methods

### Sprout seeds and bacterial strains.

Alfalfa (*Medicago sativa*, cv. unidentified) and fenugreek (*Trigonella foenum-graecum*, cv. unidentified) seeds were obtained from a commercial source (Otis S. Twilley Seed Co., Inc.) and stored at 10°C before being used in experiments. *Salmonella enterica* subspecies *enterica* serotypes Stanley and Cubana were used in the study, and these two *Salmonella* strains have been previously linked to sprout-associated outbreaks of infections (Mahon et al., 1997; Huang et al., 2019). Nalidixic acid (NA)-resistant derivates of the bacterial strains selected prior to the experiments (Cui et al., 2020) were grown on tryptic soy agar (TSA) supplemented with 50 μg/mL of NA (NA-TSA; MP Biomedicals) at 37°C for 16 h. The obtained cultures were purified on bismuth sulfite agar (BSA) (Becton, Dickinson, and Company [BD] Sparks, MD) under the same experimental conditions.

The PIM (MW: 332.43) in freeze-dried form was provided by Ascribe Biosciences Inc. The product was dissolved in 100% ethanol to make a 10 mM stock solution which was kept at −20°C before use. The stock solution was diluted using sterile distilled water to working concentrations of 1 mM or 1 μM for seed treatments.

### Preparation of lyophilized bacterial inoculums in sandy soil.

Purified *S*. Stanley and *S*. Cubana were inoculated on TSA plates and incubated for 24 h at 37°C. Single colonies were selected and transferred to tryptic soy broth (TSB) and incubated under the same condition. The resulting cultures were centrifuged at 5000 × *g* for 5 min (Brinkmann instruments. Inc.), and the supernatants were discarded. Cell pellets were washed twice with sterilized phosphate-buffered saline (PBS; pH 7.4). After the final wash, cells were resuspended in 10% sterilized skim milk (Walmart Inc.) to a final concentration of *ca*. 10^9^ CFU/mL. The cell suspensions were transferred into a glass test tube and kept at −20°C for 24 h. A Benchtop Freeze Dry system (Lab-cono) was used to freeze-dry the cell cultures. The 10% skim milk was used to prevent possible sublethal injury and loss of viability of bacterial cells.

Lyophilized cells of *S*. Stanley and *S*. Cubana (10^8^ CFU/g) were then mixed with sterilized sandy soil (Mosser Lee Co.) at a ratio of 0.1:100 to make the pathogen population at 10^4^−10^5^ CFU/g of soil. The inoculated soil was hand-massaged for 3 min, followed by agitation on an orbital shaker (model 3520, Lab-Line Instruments) at 250 rpm and room temperature for 18 h to ascertain that the inoculated bacterial cells were evenly distributed in the soil. The contaminated sandy soils were used as a vehicle to inoculate sprout seeds.

### Removal of natural microflora from sprout seeds.

A commercial sodium hypochlorite solution (6%; The Clorox Company) was diluted in sterile water at a ratio of 1:2 to reach a concentration of 20,000 ppm. Purchased alfalfa and fenugreek seeds (2 g) were treated with the diluted sodium hypochlorite (5 mL per gram of seeds) with agitation on a platform shaker (Model 421105, Nutator) for 15 min at room temperature. Spent sanitizer solution was aspirated, and residual sodium hypochlorite on alfalfa or fenugreek seeds was neutralized with 5 mL Dey-Engley neutralization broth (BD) for 10 min with agitation. The seeds were then rinsed twice with 5 mL of sterilized deionized water before being dried on a piece of sterilized weighing paper (Fisher Scientific) for 1 h in a biological safety cabinet (class II type A/B 3, Nuaire) at room temperature.

### Seed treatment with the PIM.

Sanitized alfalfa and fenugreek seeds were treated with the PIM at 1 mM or 1 μM (1.6 mL per gram of seeds). The control groups of seeds were treated with the same volumes of 10% or 0.01% ethanol that were in the working concentrations of the PIM solutions. Seeds in both experimental and control groups were treated at room temperature for 20 min. In the following, treatment solutions were removed, and treated seeds were dried on sterile weighing paper for 1 h at room temperature.

Dried alfalfa and fenugreek seeds were then mixed with inoculated sandy soil, respectively, at a ratio of 1:10 in a Whirl-Pak bag (Nasco Fort Atkinson) and agitated on an orbital shaker (Lab-Line Instruments) at 250 rpm for 1 h. The sample bags were flipped over after the first 30 min of agitation. The seeds and soil were then separated using a sterile sieve (hole size 0.4 mm), and *Salmonella* cells loosely associated with inoculated seeds and remaining sand particles on seeds were removed by rinsing twice with sterilized DI water. Subsequently, the seeds were used for sprouting and for the determination of precise levels of bacterial inoculation.

### Sprouting.

Seeds for the sprouting experiments were planted on 1% water agar (BD) in squared Petri-dishes with a 6*6 grid (Simport Beloeil). Seeds treated with the PIM were planted on 1% water agar containing 10 μM of the PIM, while the control seeds were planted on water agar without the PIM. The Petri-dishes were placed in transparent plastic containers (66Qt. Steritile) with damp paper towels at the bottom of the containers for 7 days at 25°C in the dark. On day 0 and Day 1, the seed (0.5 g) or five pieces of sprout samples of each kind with appropriate weights were mixed with 2.5 mL of PBS. While on days 3, 5, and 7, the same pieces of sprouts of each kind were weighed before being mixed with 5 mL of PBS. The samples were homogenized for 1 min using a pestle. Bacterial cell populations in the resulting homogenates were subsequently determined using both NA-TSA and BSA plates.

In the event when *Salmonella* population dropped below the detectable level of the plate count assay (detection limits: 0.88–2.05 log CFU/g of seeds/sprouts depending on sample weights at each sampling point), each sprout seed homogenate was aseptically mixed with a 9-time volume of lactose broth (BD), and the mixture was vortexed for 1 min and left at room temperature for 60 min. The pH of the mixture was adjusted to 6.8, when necessary. The samples were incubated for 24 h at 37°C; Into each sprout homogenate, universal preenrichment broth (M188) (BD) in 9-time volume was added, and the mixture was vortexed for 1 min before being incubated for 24 h at 37°C. Preenriched mixture (0.1 mL) was transferred to 10 mL Rappaport-Vassiliadis (RV) broth (BD) and another 1 mL mixture to 10 mL tetrathionate (TT) broth (BD). The RV broth was incubated for 24 h at 42°C, while the TT broth was for 24 h at 37°C. Subsequently, a loopful (10 μL of enriched cultures from the RV and TT broth was inoculated on NA-TSA and BSA. The plates were incubated for 24 h at 37°C.

### Statistical analysis.

The experiment included two seed types, two strain types, four treatment types, five sampling points, and two replicates with a total sample size of 160. Data collected were fit into the general linear model of the Statistical Analysis Software (version 9.4; SAS, Institute, Cary, NC). Fisher’s least significant difference test was used to separate the means. Significant differences in pathogen cell populations (*P* ≤ 0.05) resulted from different seed types, strain types, treatment types, and sprouting times were compared.

## Results

### Results of overall statistical analysis.

**Figure 1.**
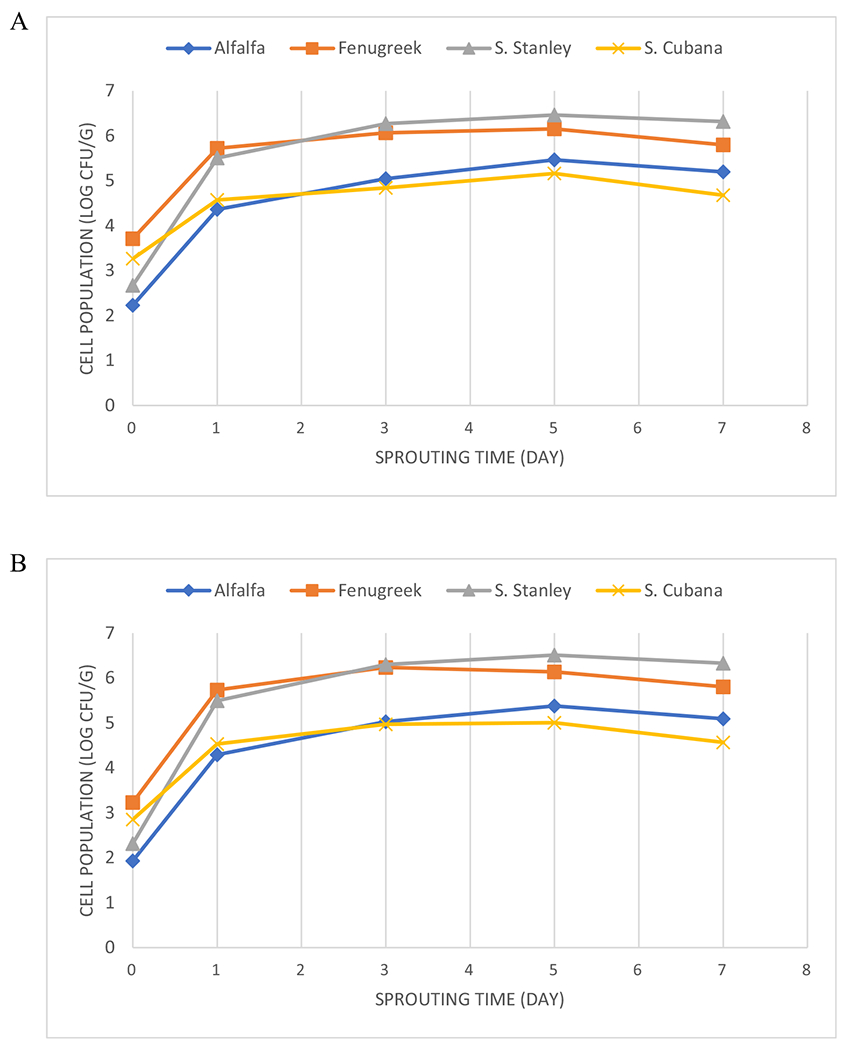
*Mean populations of cells on alfalfa and fenugreek sprouts contaminated with* Salmonella enterica *during the sprouting process. A. mean cell populations of both* Salmonella *strains on individual sprout (labeled as alfalfa or fenugreek) samples or the mean cell populations of individual* Salmonella *strains (labeled as Stanley or Cubana) from both types of sprout samples at each sampling point according to the enumeration results from NA-TSA plates. B. mean cell populations of both* Salmonella *strains on individual sprout (labeled as alfalfa or fenugreek) samples or the mean cell population of individual* Salmonella *strains (labeled as Stanley or Cubana) from both types of sprout samples at each sampling point according to the enumeration results from BSA plates*.

The four defined independent variables, seed type, strain type, treatment type, and sprouting time, were all significant (*P* ≤ 0.05) factors influencing the growth of *Salmonella* on sprouts (Table 1). There were significant interactions between ascr treatment and sprout seeds and between ascr treatment and *Salmonella* strains used in the study. The mean populations of *Salmonella* on fenugreek seeds and sprouts were significantly higher than those on alfalfa seeds and sprouts (Table 2). *S*. Stanley grew better than *S*. Cubana on seed and sprout samples. The group treated with 0.01% ethanol had a significantly higher cell population than those treated with 10% ethanol. Treatment with the PIM at 1 mM reduced the mean pathogen population by about 4.0 log CFU/g compared to the group with 10% ethanol. However, treatment with the PIM at 1 μM only reduced the mean *Salmonella* population by *ca*. 1 log CFU/g. The mean population of *Salmonella* on sprouts increased with sprouting time and reached the peak population on day 5 (Table 2 and Fig. 1).

Under the treatments with 1 mM of the PIM and 0.01% ethanol, the mean cell populations of *Salmonella* on fenugreek sprouts were significantly higher (*P* ≤ 0.05) than those on alfalfa sprouts (Table 3). However, when 1 μM of the PIM was used, opposite results were noticed. Furthermore, there was no significant population difference (*P* > 0.05) between the two types of sprouts treated with 10% ethanol.

### Interactions between treatment type and seed type.

The mean cell populations of the two *Salmonella* strains in the two treated groups were significantly lower (*P* ≤ 0.05) than the control groups regardless of the type of growth media and seeds used in the experiment (Table 3). Alfalfa sprout samples from the two controls had similar (*P* > 0.05) mean cell populations when enumerated on NA-TSA and BSA, while the fenugreek sprouts from the control group with 0.01% ethanol had a higher cell population than samples from the other control group. The average reduction in the mean *Salmonella* population resulting from the 1 mM treatments was 5.68 log CFU/g on alfalfa sprouts and 2.26 log CFU/g on fenugreek sprouts according to the enumeration results from BSA. As for the 1 μM treatment, the level reductions from alfalfa and fenugreek sprouts were 0.45 and 2.22 log CFU/g, respectively.

### Interaction between treatment type and bacterial strain type.

Like the results of the overall statistical analysis (Table 2), *S*. Stanley in general had a significantly higher (*P* ≤ 0.05) cell population than *S*. Cubana in individual treatment groups (Table 4). However, in the control group with 0.01% ethanol, the population of the two *Salmonella* strains was similar (*P* > 0.05).

Populations of the two individual *Salmonella* strains were significantly lower (*P* ≤ 0.05) in the PIM-treated groups than in the control groups (Table 4). The 1 mM treatment was more effective than the 1 μM treatment. *S*. Stanley had a higher cell population in the control group with 0.01% ethanol compared to the group with 10% ethanol. But the *S*. Cubana population was significantly higher in the group with 10% ethanol. The 1 mM treatments reduced the populations of *S*. Stanley and *S*. Cubana by 4.24 and 3.71 log CFU/g, respectively. The 1 μM treatment reduced the population of *S*. Stanley by 4.95 log CFU/g and that of *S*. Cubana by 3.72 log CFU/g according to the enumeration results from BSA.

### Detection limits and results of enrichment.

All alfalfa samples that were treated with the PIM at 1 mM and inoculated with either *S*. Cubana or *S*. Stanley tested negative for the pathogen in the plate count assay. The detection limit for *S*. Cubana contaminated samples was calculated as 1.00 log CFU/g on Day 0, 0.89–2.00 log CFU/g on Day 1, 1.25–2.00 log CFU/g on Day 3, 1.10–1.94 log CFU/g on Day 5, and 1.03–1.96 log CFU/g on Day 7, depending on the weight of the seed/sprout samples. All samples that tested negative for *Salmonella* in the plate count assay were subjected to enrichment. Results showed that only the samples on Day 5 tested positive for *S*. Cubana.

The detection limit for *S*. Stanley contaminated samples was 1.00 log CFU/g on Day 0, 0.88–2.05 log CFU/g on Day 1, 1.29–2.05 log CFU/g on Day 3, 1.16–2.02 log CFU/g on Day 5, and 1.10–2.00 log CFU/g on Day 7. With enrichment, the alfalfa seed samples treated with the PIM at 1 mM tested positive for the pathogen on Day 0, but the Day 1 to Day 7 samples treated with the same concentration of the PIM were all negative for the pathogen in the enrichment assay.

## Discussion

The chemical structure indicates that ascaroside molecules are highly lipophilic, which means they have a low solubility in aqueous media (Choe et al., 2012). For this reason, the PIM in freeze-dried form was first dissolved in 100% ethanol in this study before being diluted to work concentrations using sterile distilled water. According to a previous study, certain concentrations of ethanol negatively affected the development of plants (Sako et al., 2018), for example, 10% ethanol projected stress to, and 25% ethanol could kill, plant cells (Nicholson, 2019). Ethanol can also inhibit the germination of plant seeds. It was found in a study that 1–10 mM of ethanol inhibited the germination of tomato seeds (Chen et al., 2020). To avoid any probable impact of ethanol on seed germination, sprout development, and *Salmonella* growth, treatments with 10% and 0.01% ethanol, the concentrations of ethanol in 1 mM and 1 μM of the PIM working solutions were included in the current studies as the control groups. Since 10 μM of the PIM was added to the water agar for sprouting, it would be beneficial if water agar containing 0.1% ethanol was also included in the study as an additional control.

According to the results in Table 1, the PIM treatment is a significant main factor in reducing the population of *Salmonella* on alfalfa and fenugreek sprouts. According to our knowledge, the PIM has not been used to control human pathogen growth on sprouts, but it has been tested against plant pathogens. In a study by Manosalva et al. (2015), treatment with the PIM at 1 μM was used to treat the roots of *Arabidopsis* for 24 h before inoculation with *Pseudomonas syringae* pv. tomato (*Pst*). The treatment decreased the population of *Pst* from 6.70 to 5.60 log CFU/cm^2^ of tomato leaves. In this study, treatment with the PIM at 1 μM mediated the reduction of the population of *Salmonella* by *ca*. 1 log CFU/g of sprout (Table 2). A dose-response was found in the current study: treatment with the PIM at 1 mM was more effective than the 1 μM treatment. However, increasing the ascaroside concentration to 5 μM in the study by Manosalva et al. (2015) was less effective than the 1 μM treatment when applied as root treatment. It is not known whether a dose-response could be observed if a relatively large concentration difference was used.

The current study found that the mean population of *Salmonella* on fenugreek seeds and sprouts was significantly higher (*P* ≤ 0.05) than the population on alfalfa seeds and sprouts (Table 2). Using the same seed inoculation approach with contaminated sandy soil, a similar observation was made by Cui et al. (2020). Another study examined the growth rate of *E. coli* in the extracts of alfalfa and fenugreek sprouts, and it was noticed that all tested bacterial strains including *E. coli* MG1655 (OR:H48), JHI5025 (unknown serotype), JHI5039 (unknown serotype), Sakai (O157:H7), and ZAP1589 (O157:H7) grew faster in fenugreek extract than in alfalfa extract, especially at 25°C (Merget et al., 2019).

Bacterial colonization on seeds is determined by many factors that can impact the probability and suitability of bacterial growth. The population difference of *Salmonella* in the two types of sprouts may be attributed to their nutrient compositions. A previous study examined the contents of five different polyamines, including agmatine, putrescine, cadaverine, spermidine, and spermine, before and after alfalfa and fenugreek seed germination (Cigić et al., 2020). The mass of putrescine and cadaverine in a single fenugreek sprout was 1,079 mg/kg and 3,563 mg/kg, which were both higher than those in an alfalfa sprout, 1,015 and 1,910 mg/kg, respectively. During the sprouting process, the total mass of the five polyamines in fenugreek sprouts increased by 4,808 mg/kg, while in alfalfa sprouts, the mass of total polyamines increased by only 3,051 mg/kg. Polyamines are organic compounds having more than two amino groups, and they are necessary for the physiological functions of bacteria, including growth, biofilm formation, and the production of products vital for their survival, such as the production of siderophores (Michael, 2018).

The minimum nutrients required for bacterial growth include water, carbon source, nitrogen source, and inorganic substances such as phosphate and sulfate (Taylor, 2019). A previous study found that seeds with larger mass had more minerals and carbon-based reserves than those with smaller mass (Milberg et al., 1998). A fenugreek seed is much larger than an alfalfa seed. The average weights of a single alfalfa and fenugreek seed are 2 mg and 16 mg, respectively (Rankin, 2008). A study conducted by Vaughton and Ramsey (2001) found that the concentrations of nitrogen and phosphorus in large seeds were over 10-fold higher than in small seeds. According to the relationships between seed mass and nutrient content provided by the study, the nitrogen and phosphorus contents in a single alfalfa seed were *ca*. 100 μM and 10 μM, respectively. While the concentrations of the two nutrient components per fenugreek seed are 1,300 μM and 120 μM (Altuntaş et al., 2005). Nitrogen is an essential element of life since it is a component of amino acids and nucleotides which are necessary for the synthesis of proteins and nucleic acids (Howarth et al., 2022). Phosphorus is also essential for life because it composes the backbone of DNA and RNA, energy carrier ATP, cell membrane, and so on (Elser, 2012).

Results of the current study showed that *S*. Stanley has a significantly higher (*P* ≤ 0.05) population on seeds and sprouts than *S*. Cubana (Table 2), which is consistent with the result from Cui et al. (2020). The growth of *Salmonella* was serotype-dependent (Oscar, 2000; Juneja et al., 2003). A previous study compared the growth kinetics of some *Salmonella* serotypes and found that different serotypes had different growth paces. Among the tested strains, *Salmonella* serotype Paratyphi B reached the stationary phase within 24 h and had a peak population of 9.68 log CFU/mL at the stationary phase, while *Salmonella* serotype Typhi did not reach the stationary phase even after 48 h of inoculation and the population at 48 h was only 9.18 log CFU/mL (Díez-García et al., 2012).

*Salmonella* populations increased by about 3 log CFU/g on both types of sprouts from day 0 to day 1 (Fig. 1B). This change is consistent with the results of a previous study that used bacteriophages to control *Salmonella* growth (Pao et al., 2004). After day 1, the mean populations of the pathogen became stable at *ca*. 5 log CFU/g with a slight drop thereafter. Similar results have been also reported by Fong et al. (2017). This phenomenon could be caused by the carrying capacity of the niche being reached, which limits the growth of bacterial pathogens on sprouts after the first day of sprouting (Howard and Hutcheson, 2003).

Although treatment with the PIM at 1 mM was effective in the control of *Salmonella* on alfalfa sprouts (Tables 2 and 3), *S*. Cubana was detected on Day 5 through the enrichment assay. This finding emphasizes the challenges of controlling pathogen growth on sprouts and sprout-associated outbreaks of infections. In commercial sprout production, a large number of vegetable seeds are used. In an event when a single seed evades sanitizing treatment or plant immune response, the consequence could be devastating.

In summary, the results of this study reveal that low concentrations of the PIM tested in the study can reduce the populations of two *Salmonella* strains artificially inoculated on alfalfa and fenugreek seeds during sprouting. Treatment with the PIM at 1 mM was more effective than the 1 μM treatment. The mean populations of *Salmonella* on the seeds and sprouts are influenced by seed type, *Salmonella* strain type, treatment type, and sprouting time. The study provides supporting evidence for the potential use of the PIM for the control of pathogen contamination on edible sprouts. The PIM treatment residue test, sensory evaluation, and toxicology study are being conducted to determine the feasibility of its use for food application.

## Figures and Tables

**Table 1:** Type III tests for fixed effects by the statistical model of sprout sampling (α = 0.05)

NA-TSA					
Source	DF	Type III SS	Mean Square	F Value	Pr > F
Seed	1	42.30	42.30	164.79	<.0001
Strain	1	35.43	35.43	138.01	<.0001
Treatment	3	500.49	166.83	649.88	<.0001
Time	4	170.75	42.69	166.29	<.0001
Replicate	1	0.090	0.090	0.35	0.556
Seed*Treatment	3	108.20	36.07	140.50	<.0001
Strain*Treatment	3	22.72	7.57	29.50	<.0001
**BSA**					
Seed	1	47.08	47.08	192.11	<.0001
Strain	1	40.35	40.35	164.66	<.0001
Treatment	3	467.28	155.76	635.61	<.0001
Time	4	222.57	55.64	227.06	<.0001
Replicate	1	0.46	0.46	1.87	0.175
Seed*Treatment	3	108.89	36.30	148.12	<.0001
Strain*Treatment	3	20.65	6.88	28.08	<.0001

Type III error: correctly rejecting the null hypothesis for the wrong reason.

DF: Degree of freedom.

Pr > F: *P* value, the tested effect is significant when the *P* value is ≤ 0.05.

Sample size 160 = 2 seed types × 2 strain types × 4 treatment types × 5 sampling points × 2 replicates.

**Table 2:** Overall mean total aerobic and *Salmonella* counts at different sampling points and from alfalfa and fenugreek sprouts developed from seeds underwent different treatments

		*Salmonella* population (Log CFU/g)	
		NA-TSA	BSA
Sprout seeds			
	Fenugreek (n = 80)	5.49A	5.43A
	Alfalfa (n = 80)	4.46B	4.34B
Bacterial strain			
	*S*. Stanley (n = 80)	5.45A	5.39A
	*S*. Cubana (n = 80)	4.51B	4.38B
Seed treatment			
	EtOH (0.01%) (n = 40)	6.46A	6.36A
	EtOH (10%) (n = 40)	6.16B	6.00B
	PIM (1 μM) (n = 40)	5.27C	5.17C
	PIM (1 mM) (n = 40)	2.01D	2.02D
Sprout time			
	5 (n = 32)	5.81A	5.76A
	3 (n = 32)	5.56B	5.63AB
	7 (n = 32)	5.50B	5.45B
	1 (n = 32)	5.04C	5.01C
	0 (n = 32)	2.97D	2.58D
Replicate			
	One (n = 80)	5.00A	4.94A
	Two (n = 80)	4.95A	4.83A

Values of the same variables in each column followed by the same letters are not significantly different (*P* > 0.05). EtOH: ethanol; PIM: plant immunity modulator, an (ω – 1)-hydroxy fatty acid ascaroside excreted by plant-parasitic nematodes; NA-TSA: tryptic soy agar supplemented with nalidixic acid; BSA: bismuth sulfite agar.

Sample size: 160 = 2 seed types X 2 strain types X 4 treatment types X 5 sampling points X 2 replicates

**Table 3:** Mean total aerobic and *Salmonella* counts in samples collected from alfalfa or fenugreek sprouts developed from seeds underwent different treatments

Seed treatment		Cell population (Log CFU/g)			
		EtOH (10%)	EtOH (0.01%)	PIM (1 μM)	PIM (1 mM)
NA-TSA (n = 160)					
	Alfalfa (n = 80)	6.04Aa	6.07Ba	5.60Ab	0.14Bc
	Fenugreek (n = 80)	6.28Ab	6.86Aa	4.95Bc	3.88Ad
BSA (n = 160)					
	Alfalfa (n = 80)	6.81Aa	5.94Ba	5.49Ab	0.13Bc
	Fenugreek (n = 80)	6.18Ab	6.78Aa	4.84Bc	3.92Ad

Values within the same column followed by the same uppercase letters are not significantly different (*P* > 0.05); values within the same row followed by the same lowercase letters from the same growth medium are not significantly different (*P* > 0.05). EtOH: ethanol; PIM: plant immunity modulator, an (ω – 1)-hydroxy fatty acid ascaroside excreted by plant-parasitic nematodes; NA-TSA: tryptic soy agar supplemented with nalidixic acid; and BSA: bismuth sulfite agar.

**Table 4:** Mean S. Cubana and S. Stanley counts in samples collected from both types of sprouts developed from seeds underwent different treatments

Seed treatment		Cell population (Log CFU/g)			
		EtOH (10%)	EtOH (0.01%)	PIM (1 μM)	PIM (1 mM)
NA-TSA (n = 160)					
	*S*. Cubana (n = 80)	5.24Bb	6.42Aa	5.12Bb	1.24Bc
	*S*. Stanley (n = 80)	7.08Aa	6.51Ab	5.43Ac	2.77Ad
BSA (n = 160)					
	*S*. Cubana (n = 80)	5.02Bb	6.26Aa	4.95Bb	1.31Bc
	*S*. Stanley (n = 80)	6.98Aa	6.46Ab	5.38Ac	2.74Ad

Values within the same column followed by the same uppercase letters are not significantly different (*P* > 0.05); values within the same row followed by the same lowercase letters from the same growth medium are not significantly different (*P* > 0.05). EtOH: ethanol; PIM: plant immunity modulator, an (ω – 1)-hydroxy fatty acid ascaroside excreted by plant-parasitic nematodes; NA-TSA: tryptic soy agar supplemented with nalidixic acid; and BSA: bismuth sulfite agar.
